# Supplementary material for: Public health concerns for food contamination in Ghana: A scoping review
Source: PLoS One. 2023 Aug 10;18(8):e0288685. doi: 10.1371/journal.pone.0288685 (PMC10414628; doi:10.1371/journal.pone.0288685)
Supplement: Attachment — Submitted filename: COMMENTS TO AUTHORS_May 1.docx [file pone.0288685.s002.docx]

**COMMENTS TO AUTHORS**

Let me congratulate authors for taking time to research into such an important phenomenon. I find the following comments useful for enriching the content of the manuscript.

**Abstract**

The results section of the Abstract should be re-written in order to ensure increased clarity to the findings reported under the different categories.

**Introduction**

*“…*Yet, there is inadequate research into the public health and safety threats of contaminated foods in Ghana”

How to you substantiate this?

**Methods**

**Search Strategy Applied**

i. Was your search restricted to only English language literature?
ii. What were the other inclusion/exclusion criteria used?
iii. Within the search engine/databases, did you make use of limiters?
iv. Were Boalean operators used?
v. Did you use Word truncation?

Was a data extraction tool developed and used? What were the components of this tool? Thus, can you give a description of this tool?

How did you analyse your secondary data? Can you give some descriptions to the data analysis process.
