# Supplementary material for: Public health concerns for food contamination in Ghana: A scoping review
Source: PLoS One. 2023 Aug 10;18(8):e0288685. doi: 10.1371/journal.pone.0288685 (PMC10414628; doi:10.1371/journal.pone.0288685)
Supplement: Attachment — Submitted filename: Response to Reviewers.doc [file pone.0288685.s003.doc]

C/o Air Force Medical Center

Air Force Base

Takoradi

Western Region

GHANA

24th May, 2023

The Academic Editor

PLOS ONE

RESPONSE TO REVIEWERS

On behalf of my co-authors, I welcome the assessment report and comments of the two reviewers of the research article entitled “Public health concerns for food contamination in Ghana: A scoping review”. We find the comments very useful, objective, and progressive as they would help shape the paper and improve its relevance to the global readership of the PLOS ONE.

Accordingly, please find below our specific responses to the queries raised by the reviewers:

**Reviewer #1**

**Response:**

We very much appreciate the observations made by the reviewer and consider the queries very useful in improving the overall quality of the manuscript. Our specific responses to the queries are as follows:

**Introduction**

**Query 1:** Very good observation. Comment addressed in the manuscript.

**Query 2:** Well noted and addressed in the manuscript.

**Query 3:** By our exclusion criteria, we considered articles published prior to 2001 as too old for this review and thus excluded them.

**Query 4:**

Issue addressed.

**Query 5:** As regards how the Google search result (3,650,000) was handled, our response is follows:

The data search went through two levels. At level one, we applied words like “food” OR "contamination” OR “risk” OR “Ghana”, which is intended to gauge the volume of data available on the subject. The results from the initial search included: PubMed (235), JSTOR (1,466), ScienceDirect (59,120), ProQuest (44,177), Scopus (59,120), Emeralds Insight (227), Google scholar (91,200), Google search (3,650,000). With additional words (see Table 1) added, a second level search was conducted and returned the following: PubMed (86), JSTOR (94), ScienceDirect (245), ProQuest (211), Scopus (112), Emeralds Insight (82), Google scholar (127), Google search (405). In all, 1,362 articles were located. The search exercise spanned April 21^st^, 2022, to June 19^th^, 2022. Accordingly, the manuscript has been recast to reflect this. Moreover, a PRISMA-statement on the screening process submitted to the journal for further guidance.

**Table 1**

**Query 6:** Why “Gold Coast?”

On the use of “Gold Coast” as a key word. We uphold the observations made by the reviewer and accordingly deleted it from the manuscript.

**Query 7:** Respectfully, the articles went through a rigorous screening process and must meet the inclusion and exclusion criteria defined in Table 1. So, in addition to serials 1 and 2 of the inclusion criteria, the articles, be it grey (mainly masters’ and doctoral dissertations/theses) or published articles must provide details on food safety in Ghana covering 2001 – June, 2022; and must provide details on methodology, sample/population, study area, causes of food contamination, microbial isolation, high risk foods, prevention of food contamination, and conclusion.

**Results**

**Query 8:**

Regarding the grey literature, we were clear in our intention that grey literature would be part of the review and provided for it in the inclusion criteria in Table 1. Besides, since the results captured details of the number of published and grey literature included, we did not consider it prudent to capture that somewhere again in the methods. Regarding the source and type of grey literature, these were mainly dissertations/theses. Moreover, sources and details of all articles included in this review are provided in Table 2.

**Query 9:** We fully uphold the observations made by the reviewer and accordingly effected necessary corrections in this portion of the manuscript. Thus, we amended to read “prevalence and regional distribution of studies on food contamination”. That is, “we found that there were far more studies on food contamination in Greater Accra than in any other region in Ghana”.

**Query 10:** The results were carefully presented according to the themes and indicated the number of articles that discussed these specific themes. Consider for example the following:

**Food Safety-Related Public Health Risks in Ghana**

Considering this specific theme and covering the issue of “**weak enforcement of regulations**” – **3 articles** (Amaami et al., 2017; Seidu, 2020; Wuliyeng, 2013), “**inadequate monitoring and supervision**” – **2 articles** (Ekow, 2018; MacArthur, 2007), and “**logistical limitations and ineffective collaboration**” – **2 articles** (Amaami et al., 2017; MacArthur, 2007)…

**Microbial Quality and Foods with High Public Health Risks**

On this and covering “**bacteria: *E. coli*/faecal coliforms**” – **11 articles** (Abakari et al., 2018; Agyarko, 2021; Annor et al., 2011; Aovare, 2017; Appietu et al., 2020; Bansah, 2018; Feglo et al., 2012; Kortei et al., 2020; MacArthur, 2007; Mwini et al., 2019; Owusu, 2015), “***Staphylococcus aureus****”* – **8 articles** (Agyarko, 2021; Annor et al., 2011; Aovare, 2017; Appietu et al., 2020; Feglo et al., 2012; Kortei et al., 2020; Mensah et al., 2002; Owusu, 2015), …

**Preventive Measures against Contamination of Ready-to-Eat Food in Ghana**

On this theme and covering “**enforcement**” – **14 articles** (Abakari et al., 2018; Aglidza, 2019; Agyarko, 2021; Amaami et al., 2017; Amedewonu, 2020; Aovare, 2017; Bansah, 2018; Ghartey, 2019; Kasu et al., 2022; Konlan, 2019; MacArthur, 2007; Manko, 2018; Odonkor et al., 2020; Wuliyeng, 2013), “**public education**” – **13 articles** (Ahmed, 2018; Akabanda et al., 2017; Akonor et al., 2013; Amedewonu, 2020; Aovare, 2017; Ayensu, 2020; Ayinpokaapegyine, 2016; Kasu et al., 2022; MacArthur, 2007; Monney et al., 2014; Odonkor et al., 2020; Ovai et al., 2019; Owusu, 2015)…

Thus, the concerns raised by the reviewer have already been sufficiently addressed.

**Query 11:** This concern has been adequately addressed and catered for already. Respectfully, there is no action required on this.

**Discussion**

**Query 12:** Observation well noted and accordingly addressed.

**Query 13:** Our response to “Query 10” sufficiently addressed this observation. Both the results and discussion were organised according to the themes/objectives of the review. Therefore, no clear action required on this.

**Table 2**

**Query 14:** Table 2 broadly provides further and better details on the articles included in the review. Therefore, the table is NOT intended to provide details on themes, rather, to give readers further details on the specific articles included in the review. Moreover, what the reviewer seeks for is sufficiently provided for in the results.

**Query 15:** As already provided in response to “Query 7”, the methods is logically presented and explained to ensure ease of replicability. The articles went through adequate distillation and rigorous screening process consistent with the inclusion and exclusion criteria defined in Table 1. So, in addition to just serial 1 and 2 of the inclusion criteria, the articles, be it grey (mainly masters’ and doctoral dissertations/theses) or published, articles must provide details on food safety in Ghana covering 2001 – June, 2022; and further provide details on methodology, sample/population, study area, causes of food contamination, microbial isolation, high risk foods, prevention of food contamination, and conclusion.

**Figure 1:**

**Queries 16-20:** The Figure 1, the PRISMA diagram, was prepared consistent with the standard presentation format defined in the search strategy and PRISMA statement submitted to the journal during the first submission. Therefore, the PRISMA diagram is supposed to compliment the narrative provided under the search strategy and Table 1. On the issue of the “additional records through other sources”, these are essentially articles already in the possession of the authors and others volunteered by friends, and collated for this purpose. The search strategy provided sufficient information on the PRISMA and this is consistent with the design of scoping review, which is a bit different from systematic review. While I see reason in the observations made by the reviewer, a very broad consultation with other scoping reviews published in this very journal was conducted to ensure that the design was consistent with that for scoping review.

**Reviewer #2**

We are grateful to the review for making time to review the manuscript, and overall, we find his comments very useful and progressive. Meanwhile, our specific responses to the queries raised are as follows;

**Abstract**

We find the comments appropriate and accordingly recast portions of the results.

**Introduction**

Very good observation, and we have accordingly recast the purpose to make it fit the concerns raised.

**Methods**

i. Yes, and well captured in the search strategy on Table 1.

ii. This is addressed in the search strategy.

iii, iv, & v. Limiters, Boalean operators, and extraction guideline was developed and used during the data search. The specific words applied, the levels of search, and the number of data returned from the search provided in the search strategy, including Table 1 is a part.

Sincerely,

… **SIGNED**.…

BOTHA, NKOSI NKOSI

(Corresponding Author)
